# Impact of HIV infection on consolidative radiotherapy for non-Hodgkin diffuse large B-cell lymphoma

**DOI:** 10.1186/s13014-020-01589-1

**Published:** 2020-06-15

**Authors:** Carolina Trindade Mello Medici, Geovanne Pedro Mauro, Lucas Coelho Casimiro, Eduardo Weltman

**Affiliations:** 1 Department of Radiation Oncology, Barretos Cancer Hospital, Porto Velho, RO Brazil; 2 Department of Radiology and Oncology, Medical School of Sao Paulo University, São Paulo, Brazil; 3 School of Medicine, Universidade Nove de Julho (UNINOVE), São Paulo, Brazil; 4 Department of Radiation Oncology, Hospital Israelita Albert Einstein, São Paulo, SP Brazil

**Keywords:** Radiotherapy, Diffuse large B-cell lymphoma, HIV

## Abstract

**Objectives:**

Even though frequent, it is not known how HIV infection and treatment impact in the consolidation by radiotherapy of non-Hodgkin diffuse large B-cell lymphomas (DBCL). This article aim to assess that difference that HIV makes on radiation treatment.

**Patients and methods:**

A retrospective cohort of all DBCL patients treated with chemotherapy and consolidative radiotherapy at a single institution between 2010 and 2018 was assessed. All patients had biopsy-proven lymphoma and were included if radiation was part of the treatment and had at least 6 months of follow-up or were followed until death.

**Results:**

Three-hundred fifty-nine (359) patients were selected, with a median age at diagnosis of 57.7 years (13–90 years). Twenty-eight patients (7.8%) were HIV positive. Median follow-up was 48.0 months. Female patients were 51.3% and most had a good performance in the ECOG scale (78.8% are ECOG 0–1). Median overall survival was not reached, but mean OS was 50.1 months with 86 deaths. Median progression-free survival was 48.7 months. HIV infection had no impact on OS (*p* = 0.580) or PFS (*p* = 0.347) among patients treated with RT. HIV positive patients were more frequently staged only with CT (*p* > 0.05) with no impact on PFS (*p* = 0.191). No HIV positive patient received rituximab due to local policy restrictions and HIV positive patients were more prone to receive CHOP-like chemotherapy (*p* < 0.05), specially ones with etoposide (CHOEP). CHOP was associated with better survival (*p* = 0.015) in the overall population and in the HIV negative population (*p* = 0.002), but not in the HIV positive population (*p* = 0.982). RT toxicities were not overall more frequent in the HIV positive population (*p* = 0.567), except for fatigue (*p* < 0.05) and hematological toxicities (*p* = 0.022).

**Conclusion:**

HIV status did not influence on survival when patients were treated with consolidative radiotherapy. HIV infection was a bias on our sample for staging methods and chemotherapy regimens choices. For HIV positive patients there was an increase in fatigue and hematological toxicities of any grade with radiation.

## Introduction

The role of radiotherapy (RT) in the treatment of non-Hodgkin diffuse large B-cell lymphomas (DLBCL) has been tested over the years. After the MabThera [1] trial, radiation as a consolidative treatment was not a consensus. Compared [2] the no-inferiority between RT and no consolidative therapy in initial very low-risk DLBCL and reveal positive findings [2], even though that trial can be criticized by the low threshold given to the no-RT arm for the 5-year event free survival (EFS) and for accepting a difference of EFS between the two groups larger than the difference usually seen between low-IPI no bulky disease and high-risk disease. The upcoming results of the UNFOLDER [3] trial will cast more light in the matter and publication is awaited since the no-RT arms were prematurely closed. Advanced disease is yet another discussion. The International Lymphoma Radiation Oncology Group (ILROG) has proposed guidelines to its use [4]. Nowadays, RT is an accepted option for consolidation in DLBCL.

Since it is a consolidative therapy, it is important to address the correct indication and expected toxicities of RT in DLBCL patients. It is a consensus that not every patient should receive RT and that better knowledge of the disease and the effects of radiation is the way that must be taken to improve patients’ outcomes.

An important, yet understudied, part of DLBCL patients are infected with the human immunodeficiency virus (HIV). An important French prospective cohort has shown that survival among patients living with HIV and that are diagnosed with DLBCL are like HIV negative patients [5]. In this cohort, nevertheless, radiotherapy was not part of the treatment. Therefore, its use and indications, as its toxicities, are unknown. HIV has an important role in the toxicities in oncological treatments. Both HIV infection and highly active antiretroviral therapy (HAART) can increase sensibility to radiotherapy. That has been shown in laboratory data [6] as well as in retrospective clinical data for other cancer sites [7], but never in DLBCL. With this data, we try to improve how we treat DLBCL patients that are also people living with HIV.

## Patients and methods

All patients that were diagnosed with DLBCL and treated with radiotherapy between 2010 and 2017 were retrospectively assessed. Patients were excluded if they did not receive RT, received RT in a palliative setting with relapsed disease or did not receive RT as consolidation after first line therapy. Patients with only CNS disease who received primary CNS lymphoma treatments were also excluded. All patients had biopsied-proven DLBCL, and other histology were excluded. Patients must have 6 months follow up after the completion of RT or were followed until death. Survival was assessed from the diagnosis date. Some patients were staged with Positron-emission tomography with ^18^F-fluorodeoxyglucose (^18^F-FDG) and some with whole-body tomography (CT). Both methods were valid and their use was assessed in this population. All patients were also assessed with the International Prognostic Index (IPI) and all were re-assessed by the current classification [8].

## Results

**Fig. 1:**
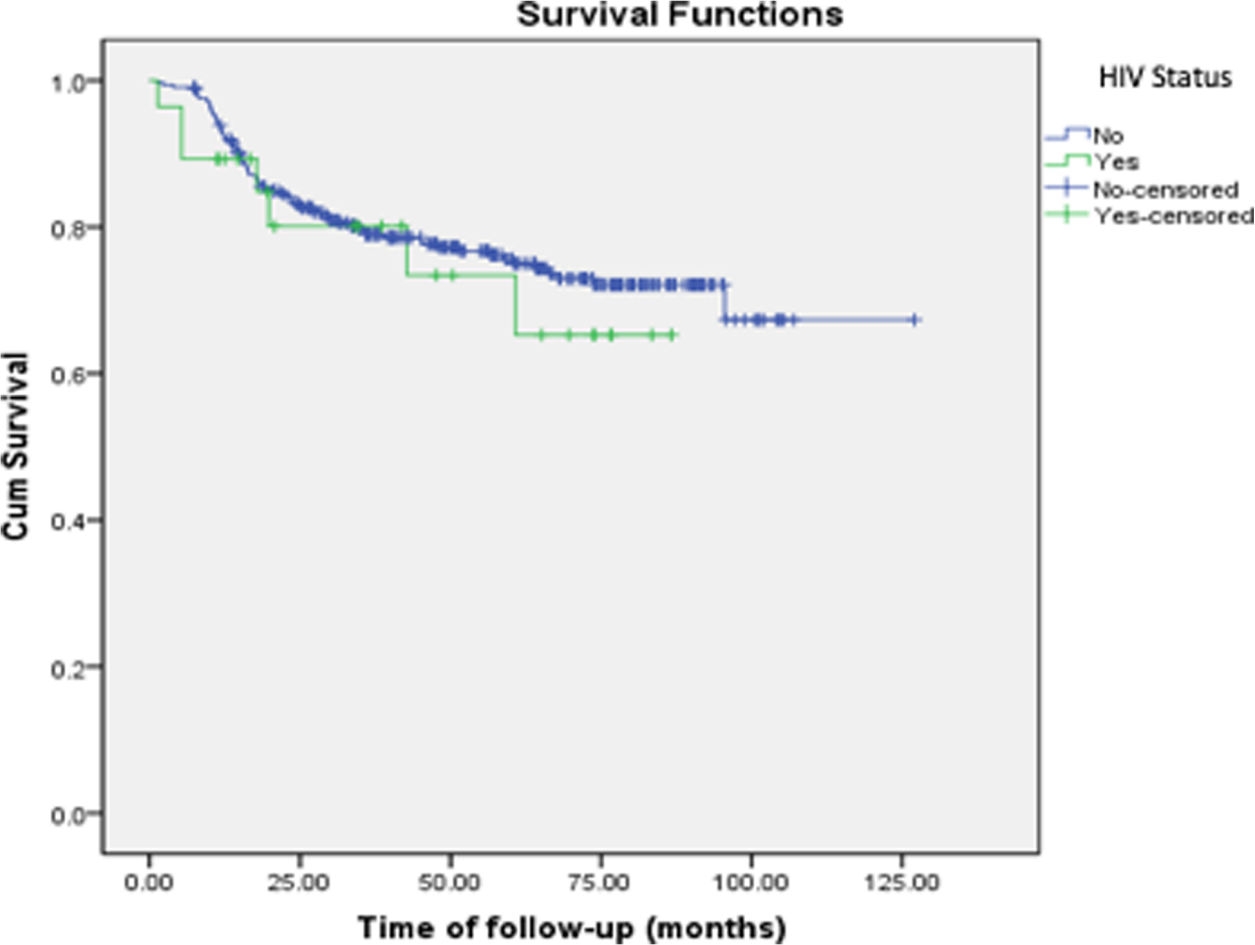
Overall Survival (by HIV status)

**Fig. 2:**
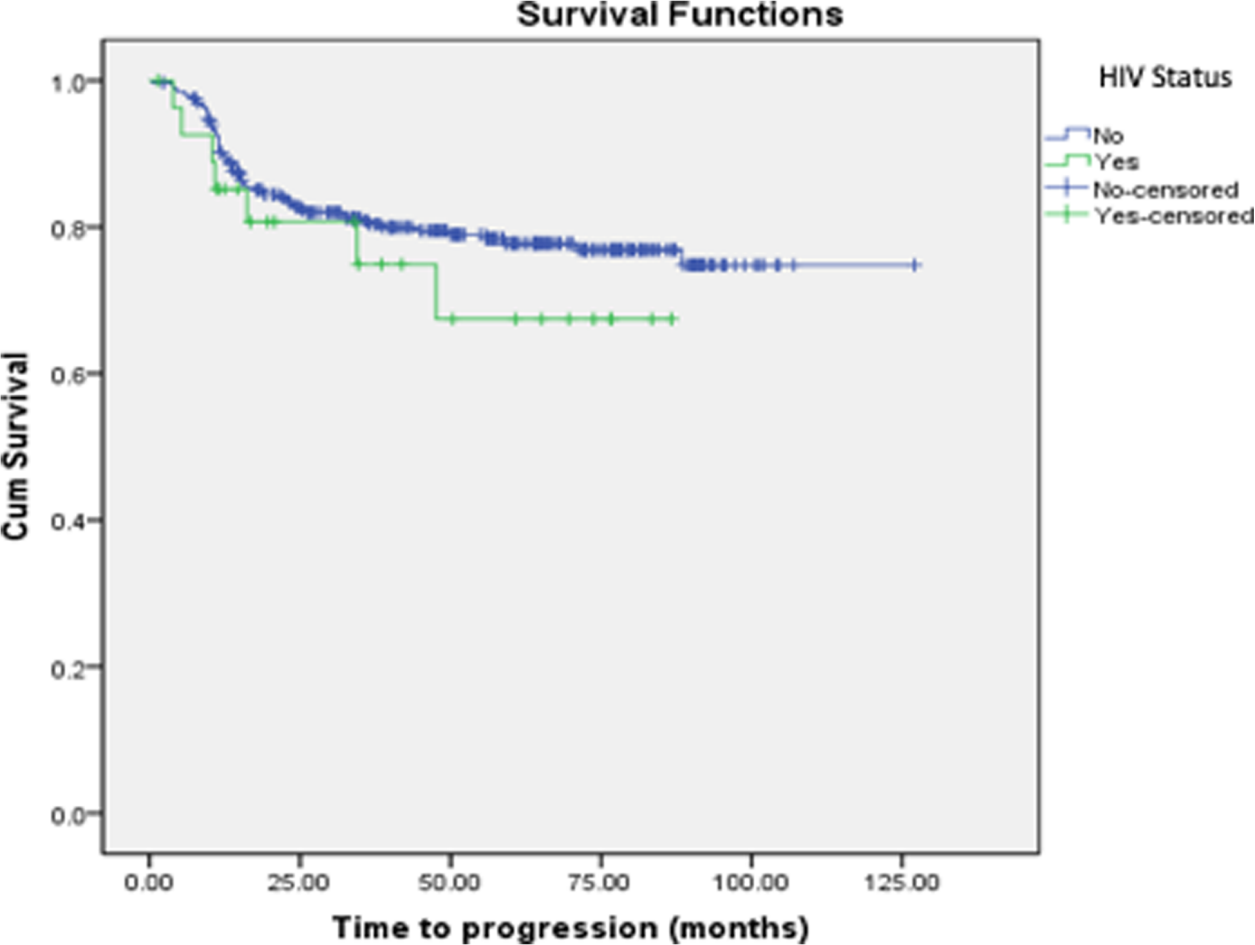
Progression-free survival (by HIV status)

There were three-hundred fifty-nine (359) patients diagnosed with DLBCL and received radiation as consolidative treatment in our cohort. Twenty-eight patients (7.8%) were people living with HIV. Most patients were female (51.3%). There was a significant difference between patients’ ages. HIV positive patients were younger than those without HIV. Gender and performance status in the ECOG scale had no difference. There was also no difference between disease first presentation localization, stage and IPI scores were well balanced between both groups. Characteristics of poor prognosis like bulky disease, extranodal disease and B symptoms were also similar between the two groups (Table 1). Median follow-up was 48.0 months. Mean overall survival was 50.1 months with no impact of HIV status (HIV negative and positive mean overall survival were 50.9 and 39.7, respectively (*p* = 0.580, Fig. 1). Mean progression free survival was also not impacted by HIV status and was 48.5 months (HIV negative and positive mean overall survival were 48.5 and 36.7, respectively (*p* = 0.347, Fig. 2). No median values were reached.

**Table 1:** Demographics

Patients characteristics	HIV		p
	No ***N*** = 331 (92.2%)	Yes ***N*** = 28 (7.8%)	
Age: mean (years)	54.4	39.0	
ECOG			
0	208 (62.8%)	20 (71.4%)	0.632
1	82 (24.8%)	3 (10.7%)	
2 or lower	41 (12.4%)	5 (17.9%)	
Staging			
PET	280 (84.6%)	10 (35.7%)	**< 0.005**
CT	51 (15.4%)	18 (64.3%)	
IPI			
Low	40 (12.1%)	3 (10.7%)	0.958
Intermediate	88 (26.6%)	8 (28.6%)	
High-Intermediate	75 (22.7%)	5 (17.9%)	
High	128 (38.6%)	12 (42.9%)	
Stage			
I	43 (13.1%)	4 (14.3%)	0.986
II	105 (31.7%)	8 (28.6%)	
III	37 (11.2%)	3 (10.7%)	
IV	145 (43.8%)	13 (46.4%)	
Localization			
Above diaphragm	111 (33.5%)	12 (42.9%)	0.593
Below diaphragm	76 (23.0%)	6 (21.4%)	
Both sides	144 (43.5%)	10 (35.7%)	
Bulky disease			
No	119 (36.0%)	11 (39.3%)	0.724
Yes	212 (64.0%)	17 (60.7%)	
Extranodal disease			
No	58 (17.5%)	6 (21.4%)	0.380
Yes	273 (82.5%)	22 (78.6%)	
B Symptoms			
No	116 (35.0%)	12 (42.9%)	0.264
Yes	215 (65.0%)	16 (57.1%)	
Chemotherapy			
CHOP	298 (90.0%)	12 (42.9%)	**< 0.005**
Others	32 (10.0%)	16 (57.1%)	
Toxicities to chemotherapy			
No toxicities	7 (2.1%)	2 (7.4%)	0.141
Grade I	41 (12.5%)	0	
Grade II	74 (22.5%)	8 (29.6%)	
Grade III	114 (34.7%)	12 (44.4%)	
Grade IV	91 (27.7%)	5 (18.5%)	
Grade V	2 (0.6%)	0	
Response to chemotherapy			
Complete response	138 (41.7%)	17 (60.7%)	0.554
Partial response	125 (37.8%)	11 (39.3%)	
Disease Progression			
No	259 (78.2%)	21 (75.0%)	0.363
Yes	72 (21.8%)	7 (25.0%)	
Death			
No	252 (76.1%)	21 (75.0%)	0.893
Yes	79 (23.9%)	7 (25.0%)

There was also no difference regarding chemotherapy outcomes. There was, nevertheless, a significant statistical difference between chemotherapy regimens. HIV positive patients received more chemotherapy based on modified CHOP (cyclophosphamide, doxorubicin hydrochloride, vincristine, and prednisone) by the addition of etoposide while the most frequent modification to HIV negative patients were the use of regimens not containing nor CHOP nor rituximab. Nevertheless, all modifications were compatible with institutional protocol. No patient living with HIV received rituximab. The results, although, were similar. No difference was seen in PFS or OS between both groups, not was any difference seen in chemotherapy response or toxicities. There was no increase on partial response rates between both groups and that did not affect radiotherapy indication.

Radiotherapy procedures were also consistent. Stage I and II patients received involved site radiotherapy and stage III and IV patients received radiation to bulky disease and extranodal disease sites or for partial response. No difference was seen between RT indications regarding HIV status. There was not difference in doses used in the treatment of these patients and, except for a small number of HIV negative patients treated in 2010, the use of involved-field (IFRT) and involved-site (ISRT) radiotherapy techniques were similar, even though there was a trend favoring the use of ISRT in HIV positive patients, maybe because of toxicities concerns in the beginning of the implementation of ISRT as the standard approach in our institution. Overall toxicities were also similar, except for two statistically significant differences in fatigue and hematological toxicities due to radiation (Table 2).

**Table 2:** Radiotherapy

Radiotherapy treatment	HIV		p
	No N = 331 (92.2%)	Yes N = 28 (7.8%)	
Dose			
Lower than 30Gy	29 (8.8%)	4 (14.3%)	0.779
30 or 30.6Gy	140 (42.3%)	11 (39.3%)	
36Gy	143 (43.2%)	11 (39.3%)	
Higher than 36Gy	19 (5.7%)	2 (7.1%)	
RT fractionation			
180 cGy/fraction	173 (52.3%)	15 (52.4%)	0.633
200 cGy/fraction	127 (38.4%)	9 (32.1%)	
Others	31 (9.4%)	4 (14.3%)	
RT technique			
EBRT	8 (2.4%)	0	0.180
IFRT	81 (24.5%)	3 (10.7%)	
ISRT	91 (27.5%)	12 (42.9%)	
Only bulky/ PR	151 (45.6%)	13 (46.4%)	
**Radiotherapy toxicities**			
Greatest toxicity grade			
No toxicity	72 (21.8%)	5 (17.9%)	0.567
Grade I	128 (38.7%)	13 (46.4%)	
Grade II	114 (34.4%)	10 (35.7%)	
Grade III	17 (5.1%)	0	
Grade IV	0	0	
Fatigue			
No	256 (77.3%)	11 (39.3%)	**< 0.005**
Yes	75 (22.7%)	17 (60.7%)	
Hematologic			
No	320 (96.7%)	24 (85.7%)	**0.022**
Yes	11 (3.3%)	4 (14.3%)	
Endocrinologic			
No	324 (97.9%)	28 (100%)	0.564
Yes	7 (2.1%)	0	
Metabolic			
No	328 (99.1%)	28 (100%)	0.783
Yes	3 (0.9%)	0	
Gastrointestinal			
No	339 (72.2%)	22 (78.6%)	0.315
Yes	92 (27.8%)	6 (21.4%)	
Infections			
No	313 (94.6%)	26 (92.9%)	0.474
Yes	18 (5.4%)	2 (7.1%)	
Lymphedema			
No	322 (97.3%)	28 (100%)	0.477
Yes	9 (2.7%)	0	
Musculoskeletal			
No	322 (97.3%)	28 (100%)	0.477
Yes	9 (2.7%)	0	
Neurological			
No	324 (97.9%)	27 (96.4%)	0.481
Yes	7 (2.1%)	1 (3.6%)	
Pain			
No	255 (77.0%)	23 (82.1%)	0.363
Yes	79 (23.0%)	5 (17.9%)	
Lung			
No	307 (92.7%)	28 (100%)	0.133
Yes	24 (7.3%)	0	
Genitourinay			
No	326 (98.5%)	27 (96.4%)	0.388
Yes	5 (1.5%)	1 (3.6%)	
Vascular			
No	326 (98.5%)	28 (100%)	0.665
Yes	5 (1.5%)	0	

HIV patients’ HAART characteristics were also assessed. There can be identified two trends: one of patients receiving first line HAART treatment in Brazil until 2017 (combination of tenofovir, lamivudine and efavirenz in a single pill) and a growing number of patients receiving the integrase inhibitor dolutegravir as part of their HAART after publication of prospective trials showing better outcomes with this approach over the previous first line treatment [9] since most patients were long time users of HAART (Table 3).

**Table 3:** HIV

	Number (n)	(%)
**HAART use**		
*No use*	2	7.1
*Starting at lymphoma diagnosis*	5	17.9
*Long-term users*	20	71.4
*No information*	1	3.6
**NRTI in current scheme**		
*Yes*	4	14.8
*No*	23	85.2
**NNRTI**		
*Yes*	17	63.0
*No*	10	37.0
**Protease inhibitors**		
*Yes*	15	55.6
*No*	12	44.4
**Integrase inhibitors**		
*Yes*	5	18.5
*No*	22	81.5
**Fusion inhibitors**		
*Yes*	*0*	0
*No*	*27*	*100*
**CCCR5 antagonists**		
*Yes*	0	0
*No*	*27*	*100*

## Discussion

The differences in treatment planning between HIV positive and negative patients must be highlighted. The use of PET-CT at staging is an important factor. All patients staged with PET were also assessed during chemotherapy with the same tool. Even though its use did not impact PFS (*p* = 0.103), it is important to state that PET-CT is the most important tool to assess response to chemotherapy and to correctly stage patients with DLBCL. Previous data has shown that comparisons of glucose uptake in HIV patients can produce false positive results [10]. Nevertheless, the correct staging is the basis for correct assessment and treatment planning. Since some of our HIV patients were staged with PET-CT (35.7%), this data must be seen with caution and radiologists must be trained so the best results can be achieved with this tool. The simple omission of PET-CT in HIV positive patients does not appear to be the best way to go.

Another difference that was addressed was the chemotherapy regimens. In our cohort, the use of etoposide was common in HIV positive patients. Its use has been based on prospective data [11]. Since rituximab is not approved in Brazil for HIV positive patients in the public health setting, it was stated that maybe adding etoposide to the chemotherapy would partially compensate the lack of the better drug, but our numbers showed that there was no difference among HIV patients with the use of etoposide (*p* = 0.982). On the other hand, regimens other than CHOP did negatively influenced the HIV negative population. Since the most common protocol change was lack of rituximab (due to autoimmune diseases, allergic reactions or lower institutional supply of the drug), it’s expected that it would have a negative impact on survival (*p* = 0.002). Rituximab is key to treating DBCL. Rituximab did have a survival impact on HIV negative patients. However, since no HIV positive patient received it, we have no data on the impact of this drug in this subset. The second most common protocol alteration was the use of regimens that do not contain doxorubicin due to concern on cardiac toxicities. Those two protocol deviations were not measured since the aim of this project was to assess HIV and RT, but literature shows that those protocol deviations can have consequences on survival [12].

The most important thing to state is that HIV did not influence outcomes. There was no significance in the influence of HIV in either PFS or OS (*p* = 0.499). The interquartile analysis of the HIV impact on OS has also shown that no relation can be make (0.312–1.763) and people living with HIV that are diagnosed with DLBCL should be treated as any other patient. Nevertheless, when RT is used as a consolidative therapy, a few steps must be given. Fatigue is more common, so patients should be oriented in that way. Hematological toxicities were also more common, so radiation oncologists should be aware of that while in review appointments with those patients. Since most of our patients were treated accordingly to national HAART protocol, no relation between any drug and any specific toxicity could be assessed, so suspending or changing HAART drugs during oncological treatment should not be advised regarding radiotherapy. Larger radiation fields are becoming rarer when ISRT technique is employed and changing contour and volumes outside of guidelines in HIV positive patients also should not be done. The use of smaller fields, with ISRT or involved node (INRT) should be favored in HIV positive patients since those toxicities depend on total irradiated volume. Our data shows that enhancing quality of radiation and patient assessment can make people living with HIV and are diagnosed with DLBCL have the same outcomes as HIV negative patients.

## Conclusion

HIV has some influence on DBCL treatment and our results show good quality novel data on the matter. In our sample, it influenced both staging tools and chemotherapy chosen to treat DBCL patients. In also has influenced RT toxicities, with HIV positive patients being more prone to fatigue and hematological toxicities. It did not, however, impact on RT outcomes and survival. Prospective research should be done with DBCL that are living with HIV.

## Data Availability

Data on this research is available on request to the corresponding author.

## Abbreviations

^18^F-FDG: ^18^F-fluorodeoxyglucose
CNS: Central nervous system
CT: Computerized tomography
CHOEP: Chemotherapy scheme with cyclophosphamide, doxorubicin, etoposide, vincristine and prednisone
CHOP: Chemotherapy with cyclophosphamide, doxorubicin, vincristine and prednisone
DBCL: Diffuse large B-cell lymphoma
ECOG: Eastern Cooperative Oncology Group
EFS: Event-free survival
HAART: Highly active antiretroviral therapy
HIV: Human immunodeficiency virus
IFRT: Involved-field radiotherapy
ILROG: International Lymphoma Radiation Oncology Group
IPI: International Prognostic Index
ISRT: Involved-site radiotherapy
OS: Overall survival
PFS: Progression-free survival
RT: Radiotherapy
